# Keloid: a rare clinical image

**DOI:** 10.11604/pamj.2024.49.97.45647

**Published:** 2024-11-27

**Authors:** Mayank Rai, Sadhana Misar Wajpeyi

**Affiliations:** 1 Department of Kayachikitsa, Mahatma Gandhi Ayurveda College, Hospital and Research Centre, Salod (H), Datta Meghe Institute of Medical Sciences (DU), Sawangi, Wardha, India

**Keywords:** Biopsy, fibrous, wound

## Image in medicine

At the location of a healed skin injury, keloids are an overgrowth of granulation tissue (collagen type 3), which is gradually replaced by collagen type 1. Keloids are glossy, fibrous nodules or firm, rubbery lesions that can range in color from red to black or pink to the color of the person's skin Brown in hue. The average keloid scar is greater than the actual wound. They might take weeks or months to develop completely. They are most commonly found on the chest, shoulders, earlobes, and cheeks. A 40-year-old male came with complaints of a lumpy or ridged area of skin with a pinkish color that was painless along with itching on the chest region for 8-9 months. On clinical and microscopic examinations, a biopsy was done. The patient had normal blood count and glucose levels. Through this, it was diagnosed as keloid. As per Ayurved local application of alkaline preparation was given and the patient was kept on conservative treatment for 3 months. After 1 month of follow-up, there was no itching and the keloid became dry, and blackish with a reduction in size. This image could be useful for differential diagnosis between hypertrophic scar, dermatofibromas, and keloid.

**Figure 1:**
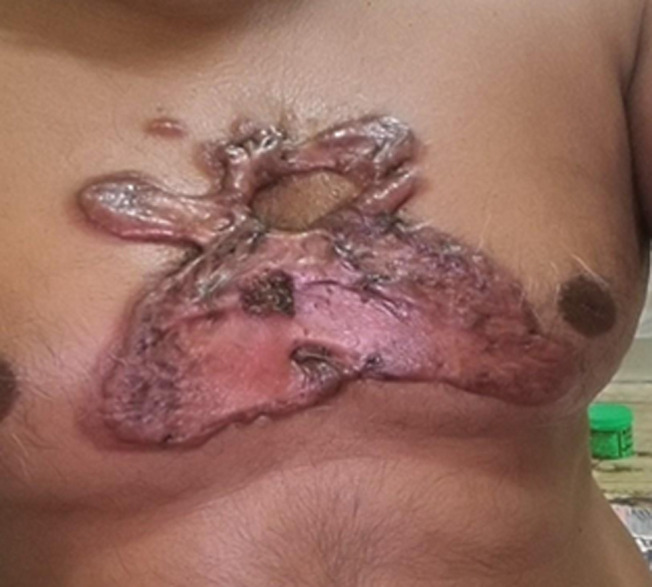
keloid scar

